# The Choice of Medicine

**Published:** 1912-09-07

**Authors:** 


### The Hospital

The Workers' Newspaper of Administrative Medicine and Institutional Life, Administration, National Insurance and Health.

No. 1366, Vol. LII. SATURDAY, SEPTEMBER 7, 1912. PRICE ONE PENNY.

### EDUCATIONAL NUMBER.

### THE CHOICE OF MEDICINE.

In his thoughtful letter to Marcellin Berthelot, Ernest Renan, in retrospective mood, confesses how his mind was clouded with doubt as to the excellence of the selection he had made in the matter of his life's work. "Ici, au bord de la mer, revenant à mes plus anciennes idées, je me suis pris à regretter d'avoir préféré les sciences historiques à celles de la nature, surtout à la physiologie comparée," he writes. Physiology was his first love, fondled at Issy and forsaken at Saint Sulpice, and ever and again his thoughts went back to it. Somewhat similar doubts may cloud the happiness of the student who has finally selected medicine as his life's work. The preliminary struggle is hard: to some minds, yearning for the larger scope of ward and bedside labour, it may even be distasteful. Chemical formulæ have no special attraction for those who do not wish to be chemists; the dog-fish and the earthworm are hardly creatures with whom one can chum unless one means to make biology the chief aim in life, and even when these preliminary difficulties have been overcome, there is enough to shadow the picture.

What does the budding medical man need? What are the requirements wanted for a medical student? A recent and much-read writer, himself a surgeon of established reputation, has laid down certain indispensable essentials, among which he particularly instances "a knowledge of Latin, a good public school education, good health, and enthusiasm." These, indeed, are indispensable, although it is true that some may cavil at the Latin and think no harm of the doctor who writes "aqua ad" in his prescriptions, or who does not know the Roman equivalent for "half a dessert-spoonful." Holmes was lenient with those who knew no Latin: when the old gentleman stated that he knew no French except pommes de terre, the autocrat said the quotation was old Italian no longer spoken. Probably a similar stepping-stone stands for any medical student who cannot read Celsus. But good health, a good average education, and enthusiasm cannot be cavilled at. They are the irreducible minimum, and without them the student cannot hope to shine. The Preliminary Arts Examination may, in a measure, be held to safeguard the second. Yet it is well that the student should bear in mind that education means infinitely more than the capacity to pass the Preliminary Arts, or even to captain a public school. It is not a means to an end: it is an end in itself. "I am a student all my days," said Paracelsus, and the emblem of the student is Linacre, from whom (probably) Browning drew his picture of the calculus-racked grammarian. As medicine advances and the profession becomes more liberal, the need for education in its true sense becomes more apparent. The doctor of to-day must be more than encyclopædic: he must possess the ability to be original and to make his originality open to all men. In the busy routine of professional life there is little opportunity to make up for lost time. What can be garnered in the student days endures, and whoever has chosen medicine must see to it that, in the beginning at least, the allurements of medical science do not wholly captivate him to the exclusion of all else besides.

Good health is an essential without which no one has the right to embark upon so arduous and exacting a career as that of the doctor. It is during his student days that the medical man will test his health and strength, and if the foundations are weak it is probable that in the dissecting-room and *post-mortem* department, in the wards and laboratories, he will lay down faults in the superstructure that in after-life will prove disastrous. The toll that ward and laboratory and hospital work exacts is greater than many imagine, for it is wearying work, carried out, often, in surroundings that are the reverse of healthy. When there is added to the difficulties arising from infirmity of body the worries of financial care, the student has a much more arduous task before him. In such circumstances only the strongest and most enthusiastic can go through the course with hope of final success. And often that success means merely the ability to register as a broken man who can, indeed, support life, but has little else available. For the qualified doctor there is always the chance of making a living.

In this respect at least the profession of medicine is superior to that of law, of navigation, of the church, or of the press. But to hold it out as an inducement to the student is to flash false promise of security before him. Without a sound constitution, even enthusiasm, be it ever so bold, can achieve little, for the dangers in the way are too great to be disregarded, and the hope of over-passing them without failure is infinitesimal. Yet enthusiasm is a very necessary quality for the medical student. Enthusiasm not only for the study and practice of medicine, but a measure as well of that general enthusiasm that transcends the difficulties of the moment and makes a mind as painfully introspective as Lamb declared his to have been sensible to the reality of what the Germans style Lebensglückseligkeit. How awful are the shocks to which such enthusiasm will be exposed has been pathetically told by Emin Pasha in his letter to his friend, Dr. Felkin. Yet Emin felt that true enthusiasm could survive all these failures and disappointments, and give, in the end, a solid satisfaction perfect in itself. It is for the student to try and gauge in some degree the nature of these perils to which his choice exposes him. In the recently published English translation of a well-known Russian doctor's "Confessions" some of these difficulties are forcibly set out, and we recommend the perusal of this interesting narrative to all medical students, not because we agree with all the conclusions drawn by the over-sensitive author, but simply because the book shows some of the shadows of professional life and work in a barefaced, perhaps even in an exaggerated manner, which comes as a welcome counter-irritant to the laudatory expositions which greet the student when he enters his medical school.

The choice once made, the future lies largely in the hands of the student himself. Tact and judgment he will learn to value on his own account, for without them he cannot progress in his school. The curriculum is framed so as to give the average student full time to qualify within six years. The enthusiastic student can easily fit himself for qualification in four years, and spend the remaining twelve months intervening between his compulsory subjects and the final in tackling special matters. Before qualifying he cannot obtain a responsible house position, but in many hospitals he can obtain a senior appointment that will give him both responsibility and experience. All hospitals do not offer these senior posts, and the student will do well, therefore, to select a school which is attached to a hospital that does offer them. Moreover, he should make a point of familiarising his seniors on the staff with his work and his character. Only by doing so can he win friends and support that will be of incalculable value to him in after-life. Subservience to authority, within limits, must be his motto while in the pupil stage; and one enemy made during this period may prove highly unprofitable to him when he applies for a post on the staff of his hospital in the future.

